# Engineering broad-spectrum digestion of polyuronides from an exolytic polysaccharide lyase

**DOI:** 10.1186/s13068-016-0455-8

**Published:** 2016-02-24

**Authors:** Logan C. MacDonald, Elizabeth B. Weiler, Bryan W. Berger

**Affiliations:** Program in Bioengineering, Lehigh University, B320 Iacocca Hall, 111 Research Drive, Bethlehem, PA 18015 USA; Department of Chemical and Biomolecular Engineering, Lehigh University, B320 Iacocca Hall, 111 Research Drive, Bethlehem, PA 18015 USA

**Keywords:** Alginate, Polysaccharide, Alginate lyase, Uronic acid, *Stenotrophomonas maltophilia*

## Abstract

**Background:**

Macroalgae represents a promising source of fermentable carbohydrates for use in the production of energy efficient biofuel. The primary carbohydrate in brown algae is the uronic acid-containing alginate, whereas green algae contains a significant amount of glucuronan. A necessary step in the conversion of these polyuronides to bioethanol is saccharification, which can be achieved by enzymatic or chemical degradation.

**Results:**

Polysaccharide lyases are a class of enzymes which cleave uronic acid-containing glycans via a β-elimination mechanism, acting both endo- and exolytically on their substrates. In the present work, we characterize a putative alginate lyase from *Stenotrophomonas maltophilia* K279a (Smlt2602) and describe a H208F mutant that, in addition to cleaving alginate-based substrates, displays significant, exolytic glucuronan activity.

**Conclusions:**

To our knowledge this is the first polysaccharide lyase to act exolytically on glucuronan and is an attractive candidate for the broad-spectrum digestion of polyuronides into fermentable monomers.

**Electronic supplementary material:**

The online version of this article (doi:10.1186/s13068-016-0455-8) contains supplementary material, which is available to authorized users.

## Background

Rising energy costs, diminishing fossil fuel resources, and increased greenhouse gas emissions have magnified the need to produce environmentally friendly biofuels via the microbial fermentation of readily available biomass. Macroalgae is a promising biomass source due to its cultivation requiring no arable land or fresh water, minimizing any economical cost due to loss of farmland currently dedicated to food production [1]. Algae is also capable of sequestering significant amounts of carbon from the atmosphere and metabolizing nutrients found in industrial effluents and municipal wastewater [2]. Furthermore, the lack of lignin in algae allows for the simple and inexpensive extraction of polysaccharides [3]. The primary carbohydrate in brown algae is alginate, whereas green algae contains significant amounts of glucuronan and its derivatives [3, 4]. Alginate is a linear copolymer comprised repeating units of d-mannuronic acid (ManA) and l-guluronic acid (GulA) arranged in three block types: repeating units of poly-β-d-ManA, repeating units of poly-α-l-GulA, and alternating units of ManA and GulA (poly-MG). Glucuronan is a homopolymer of d-glucuronic acid (GlcA), the C2 epimer of ManA [5]. Saccharification of these uronic acid-containing polysaccharides is a necessary step in their conservation to bioethanol [6].

Carbohydrate modifying enzymes offer a promising approach to produce fermentable monomers [7]. Polysaccharide lyases (PLs) are a class of carbohydrate modifying enzymes which cleave uronic acid-containing polysaccharides via a β-elimination mechanism, resulting in the formation of a double between C4 and C5 of the sugar group at the new nonreducing end [8]. This unsaturated product can be detected by monitoring absorbance at 235 nm [9]. Successful high-yield recombinant expression and purification, ease of product detection, and selectively for uronic acid monomers, which are found in many difficult to ferment glycans, make this class of enzymes advantageous for polysaccharide processing [5, 10, 11]. Previously, we reported an endolytic polysaccharide lyase (Smlt1473) from *Stenotrophomonas maltophilia* K279a that exhibited a unique pH-sensitive substrate specificity that could be significantly modified through the mutation of residues located in the active site cleft but not directly involved in the catalytic process. We demonstrated that single-point mutations H221F and R312L resulted in increased activity and specificity toward poly-ManA and poly-GlcA, respectively. These and other residues were selected based on sequence alignment and homology modeling and were hypothesized to participate in substrate binding and the precise positioning of the cleavable glycosidic bond with respect to the catalytic tetrad [5, 12].

In the present work, we characterize an exolytic polysaccharide lyase (Smlt2602) from *S. maltophilia* K279a and undertake a similar approach. Wild-type Smlt2602 (WT) acts on alginate and each of its block structures, with highest activity toward poly-ManA. Mutation of putative catalytic residues reveals that Tyr^264^ and His^418^ likely play the role of the general acid and base, respectively. Interestingly, we isolated a H208F mutant that exhibits significant exolytic activity toward poly-GlcA, a non-alginate-based substrate that Smlt2602 is virtually inactive against. Thus, unlike Smlt1473 in which substrate specificity was only modified, we are able to engineer significant novel activity into Smlt2602 via a sequence and homology model-based approach. To our knowledge, this is the first characterized bacterial lyase to demonstrate exolytic alginate and poly-GlcA activity and is another example of the structural plasticity in certain polysaccharide lyases which could be applied to the design of highly active and specific enzymes for use in glycan saccharification.

## Methods

### Homology modeling

The exolytic alginate lyase Alg17c (Protein Data Bank 4OJZ) from *Saccharophagus degradans* 2–40 was chosen as a template for constructing a homology model of Smlt2602 due to having the highest sequence identity (50 %) among polysaccharide lyases with solved crystal structures [13]. The model was built in SWISS-MODEL [14–16], and all images were generated in PyMOL [17].

### Subcloning, expression, and purification

Unless otherwise stated, standard molecular biology techniques were used for subcloning and site-directed mutagenesis [18]. An *E. coli* codon-optimized nucleotide sequence of *smlt2602* (NCBI GeneID 6,393,623) was subcloned into pET28a(+) (Invitrogen) as a BamHI-XhoI insert. Mutagenic primers were designed via PrimerX (bioinformatics.org/primer), and point mutations were generated via QuikChange II Site-directed Mutagenesis kit (Agilent Technologies). Mutations were confirmed by DNA sequencing (GeneWiz). Expression and purification were carried out in *E. coli* BL21(DE3) cells as described previously [5]. Fractions were assayed for protein content via Bradford reagent (Bio-Rad), SDS-PAGE, and immunoblotting as described previously [5]. Samples containing purified Smlt2602 were pooled together and dialyzed against 4 L of 20 mM sodium phosphate buffer (pH 8.5), 100 mM NaCl, 5 % *v/v* glycerol, 20 mM imidazole for 20 h at 4 °C, and then 20 mM sodium phosphate buffer (pH 8.5) for 40 h at 4 °C with one buffer exchange. Protein concentration was determined via absorbance measurements at 280 nm and molar extinction coefficients estimated from primary amino acid sequences [19].

### Polysaccharide substrates

Low viscosity sodium alginate and heparin were obtained from Sigma-Aldrich. Chondroitin sulfate and poly-GalA were obtained from Alfa Aesar. Hyaluronic acid, potassium salt from human umbilical cord, and heparin sulfate were obtained from MP biomedicals and sagent pharmaceuticals, respectively. Poly-GlcA was prepared from Avicel PH-105 NF (FMC Biopolymer) and its structure confirmed by ^1^H NMR as described previously [5]. Poly-ManA, poly-GulA, and poly-MG were prepared via partial acid hydrolysis of sodium alginate, and structure was confirmed by ^1^H NMR as described previously [5]. All prepared polysaccharide samples were dialyzed against 4 L of ddH_2_O for 40 h at 4 °C with one buffer exchange, lyophilized, and stored as powders at 4 °C until needed.

### Enzyme activity assays

The β-elimination mechanism of polysaccharide lyases creates a double bond between C4 and C5 of the sugar ring at the newly formed nonreducing end whose accumulation can be monitored by measuring the change in absorbance at 235 nm [9]. Absorbance measurements were taken in 1 s intervals over the course of at least 5 min via an Ultrospec 3300 UV–Vis spectrophotometer with a detection limit of 0.001 absorbance units at 235 nm per minute, as described previously [5]. One unit of enzyme activity was defined as an increase in absorbance at 235 nm of 1.0 per minute at 25 °C (1 unit = 1 ΔA_235 nm_ min^−1^) [5, 10]. In general, 3.5 μg of purified wild-type and mutant Smlt2602 was added to a solution containing 1 mg/mL substrate in a final volume of 350 μL. The pH of all reactions was controlled via specific buffers for a given pH range at a total ionic strength of 20 mM (acetate for pH 4–6, phosphate for pH 6–8.5, Tris for pH 7–9, and glycine for pH 9–10). Additionally, the Michaelis constant (*K*_*M*_) and turnover number (*k*_CAT_) of wild-type Smlt2602 and H208F mutant against alginate, poly-ManA, poly-GulA, poly-MG, and poly-GlcA were determined by varying substrate concentration. An extinction coefficient of 6150 M^−1^ cm^−1^ at 235 nm was used to convert absorbance values to product concentration [11, 12]. Initial rates (*v*_*i*_) were fit to the Michaelis–Menten equation, *v* = *k*_CAT_*E*_*0*_*S/*(*K*_*M*_ + *S*) with a generalized reduced gradient (GRG2) nonlinear optimization program [20]. The *R*^*2*^ and correlation values for each enzymes against each substrate were greater than 0.989 and 0.993, respectively. All reactions were carried out in triplicate and error is reported as standard deviation. Lyase activity was independently determined via the thiobarbituric acid (TBA) method as described previously [12, 21].

### Circular dichroism

Enzyme samples (300 μL) at 250 μg/mL in 20 mM sodium phosphate buffer (pH 8.5) were added to 1-mm path length quart cuvettes (Starna), and ellipticity was measured from 190 to 260 nm in a J-815 circular dichroism spectrometer (JASCO) at a scan speed of 100 nm/min with three accumulations per sample.

### High-performance liquid chromatography (HPLC) of unsaturated sugar products

Size-exclusion chromatography experiments were performed on an Agilent 1100 series HPLC value system equipped with a 96-well autosampler and UV–visible detector. Wild-type and mutant Smlt2602 at a final concentration of 10 μg/mL was added to poly-ManA or poly-GlcA at a final concentration of 1 mg/mL in 20 mM sodium phosphate buffer, pH 8.5, and incubated for 6 h at room temperature. The reaction mixture (15 μL) was injected into a TSKgel SuperOligoPW column (Tosoh) equipped with corresponding guard column. The mobile phase was 20 mM sodium phosphate buffer (pH 8.5) plus 250 mM NaCl and the flow rate was 0.3 mL/min. Unsaturated uronic acid products were detected by absorbance at 235 nm. Polyethylene glycol (molecular weight 600, 1000 and 2000; Alfa Aesar), d-glucuronic acid (Acros Organics) and xylose (Spectrum) were used as molecular weight standards to generate a calibration curve.

## Results and discussion

### Substrate Specificity Analysis Revealed that Smlt2602 is an Alginate-Specific Lyase

**Fig. 1:**
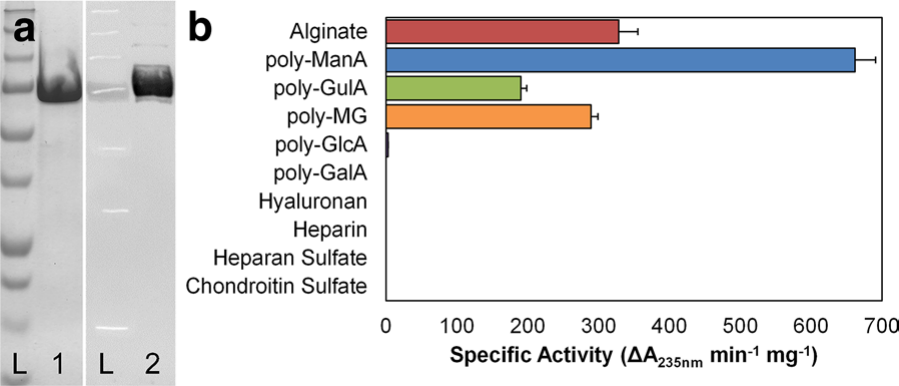
Purification and substrate specificity of WT. **a** SDS-PAGE and immunoblot of wild-type Smlt2602. *Lane L*: Precision *Blue* plus protein marker (Bio-Rad). *Lane 1*: SDS-PAGE of purified wild-type Smlt2602. *Lane 2*: Anti-His_6_ immunoblot of purified wild-type Smlt2602. **b** Specific activity of WT against 1 mg/mL of the indicated substrate in 20 mM sodium phosphate buffer, pH 8.5. Enzymatic activity was monitored by change in absorbance at 235 nm. All reactions were performed in triplicate and error is reported as standard deviation

An *E. coli* codon-optimized nucleotide sequence of *smlt2602* was subcloned into pET28a(+) as a BamHI-XhoI insert without a stop codon, resulting in the recombinant lyase containing a C-terminal His_6_ tag. Expression was carried out in *E. coli* BL21(DE3) cells at 18 °C, and Smlt2602-His_6_ was purified by passing soluble cell lysate over a Ni^2+^-bound chelating Sepharose column as described previously [5]. Approximately 60 mg of enzyme was purified per liter of induced cultured. An SDS-PAGE and anti-His_6_ immunoblot (Fig. 1a) confirmed the presence of purified Smlt2602 at the expected molecular weight of 80.8 kDa, predicted from the primary amino acid sequence of Smlt2602-His_6_ without signaling peptide [19].

The enzymatic activity of purified Smlt2602 was tested against the following ten polyuronides: alginate, poly-ManA, poly-GulA, poly-MG, poly-GlcA, poly-α-d-galacturonic acid (poly-GalA), hyaluronan, heparin, heparan sulfate, and chondroitin sulfate. Of these substrates, significant activity was measured for alginate and each of its block structures (Fig. 1b; Table 1) at an optimal pH of 8.5 (see Additional file 1: Figure S1 for relative activity against poly-ManA as a function of pH). The highest overall specific activity (661.9 ± 29.1 units/mg) was for poly-ManA, which is among the highest reported for exolytic poly-ManA degradation [22–24]. Smlt2602 exhibited approximately half the activity toward alginate (328.3 ± 27.0 units/mg) and poly-MG (289.3 ± 9.8 units/mg) and the lowest activity toward poly-GulA (190.3 ± 8.3 units/mg). No product formation was detected for the remaining substrates with the exception of poly-GlcUA, toward which Smlt2602 displayed minimal yet measurable activity (2.8 ± 0.4 units/mg, less than 0.5 % that of polyManA) (Fig. 1b; Table 1).

**Table 1:** Kinetic parameters of WT and H208F mutant

	WT			H208F		
	*k*_CAT_ (s^−1^)	*K*_*M*_ (mM)	*k*_CAT_/*K*_*M*_	*k*_CAT_ (s^−1^)	*K*_*M*_ (mM)	*k*_CAT_/*K*_*M*_
Alginate	34.8	0.67	52.2	14.6	1.35	10.8
PolyManA	62.2	0.41	151.8	21.3	0.41	51.5
Poly-GulA	34.6	5.99	5.8	1.4	2.22	0.7
Poly-MG	22.2	0.57	38.7	2.0	0.98	2.1
Poly-GlcA	0.4	1.40	0.3	6.1	0.57	10.6

WT and H208F mutant were added to 10 different solutions containing 7.8–4000 µg/mL substrate in 20 mM sodium phosphate buffer at pH 8.5 for alginate-based polysaccharides, and pH 8 for poly-GlcA. Enzymatic activity was monitored by absorbance at 235 nm, and all reactions were performed in triplicate. Turnover rate (*k*_CAT_) was calculated using an extinction coefficient of 6150 M^−1^ cm^−1^ for the unsaturated product [11, 12]. *K*_*M*_ values are based upon an effective molecular weight of 176 for the monosaccharide unit, the smallest product formed by Smlt2602 during catalysis [12, 52]

### Identification of putative catalytic and substrate-binding residues via homology modeling

**Fig. 2:**
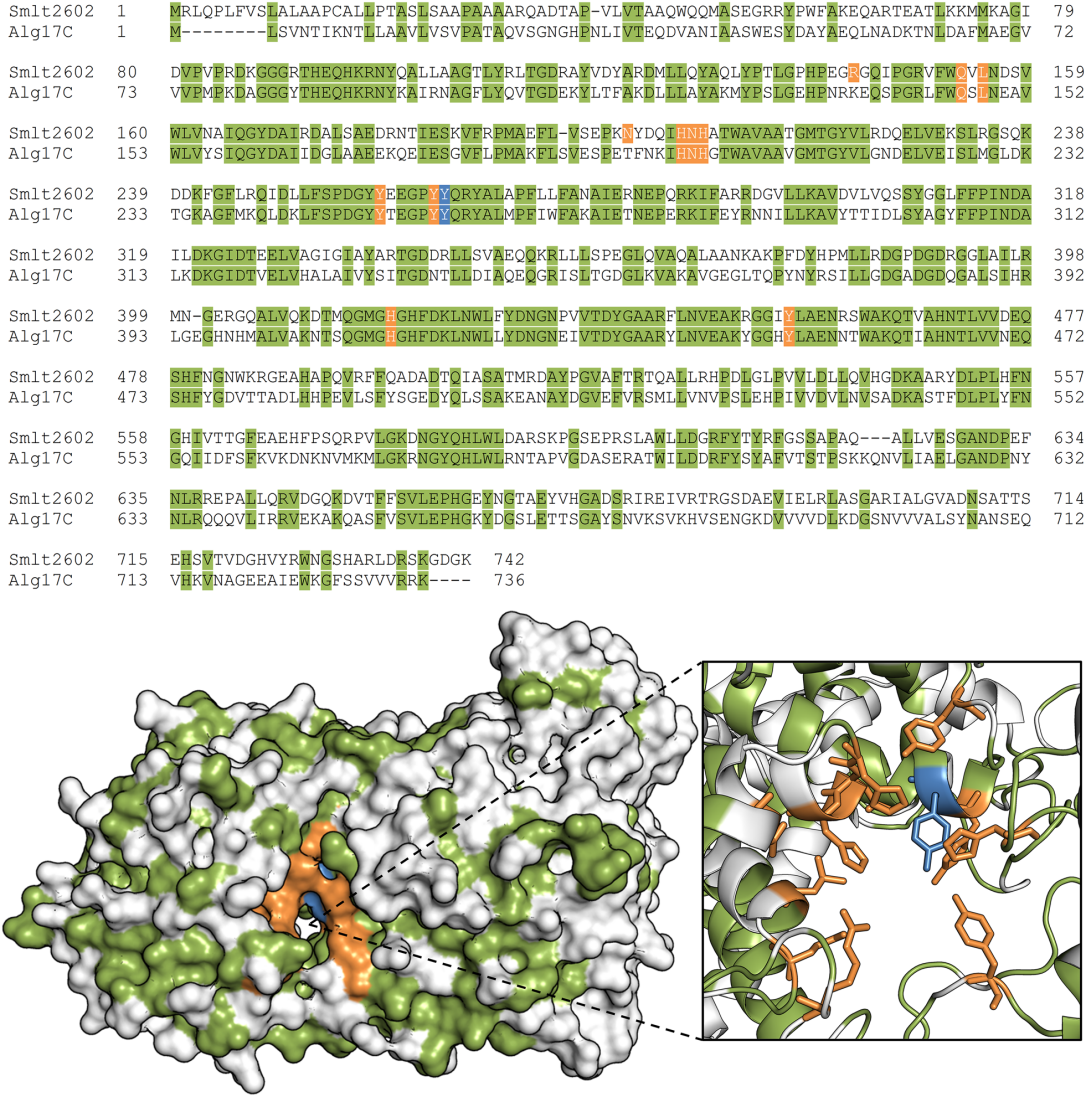
Identification of putative catalytic and substrate-binding residues in Smlt2602. Sequence alignment (*top*) and homology model (*bottom*) of Smlt2602 with *S. degradans* Alg17c lyase (Protein Data Bank 4OJZ) [13]. Identical residues are highlighted in *green*. The tyrosine residue predicted to act as the general acid in the β-elimination mechanism is highlighted in *blue* [13]. Residues predicted to be located near the active site cleft and to participate in either the catalytic mechanism or substrate binding are highlighted in *orange*

Polysaccharide lyases are divided into 22 families based on sequence, with the structure of at least one protein in each family determined. Comparison of solved crystal structures reveals that lyases from different families contain vastly different secondary structure content and thus overall fold [8]. Smlt2602 is assigned to the PL-17 family, along with Alg17c from *Saccharophagus degradans* 2–40, whose structure has recently been solved and revealed to contain two domains: an N-terminal imperfect α-barrel and C-terminal β-sheet domain [13, 25]. The circular dichroism spectrum of purified Smlt2602-His_6_ is indicative of a protein containing both α-helices and β-sheets (see Additional file 1: Figure S2 for CD spectra comparing wild-type and mutant Smlt2602). Prediction of the secondary structure from the measured spectrum estimated that Smlt2602-His_6_ is 30 % α-helical and 25 % β-sheet [26], in close agreement with the secondary structure content of Alg17c (39 % α-helical and 26 % β-sheet) [13]. Furthermore, Smlt2602 shares 50 % sequence identity with Alg17c (Fig. 2, top). Given that Smlt2602 contains the expected secondary structure content for a polysaccharide lyase belonging to the PL-17 family and exhibits significant sequence homology with Alg17c, another member of the same family, the high-resolution crystal structure of Alg17c (Protein Data Bank 4OJZ) was selected as the template to construct a homology model of Smlt2602 (Fig. 2, bottom).

In spite of diverse substrate specificity, secondary structure content, and tertiary folds, the β-elimination mechanism of PLs is remarkably conserved across the 22 families. The reaction proceeds via three chemical steps: (i) neutralization of the negatively charged C5 carboxylate group on the +1 sugar ring, reducing the p*K*_*a*_ of H5, thereby facilitating its abstraction; (ii) removal of H5 by a general base; and (iii) electron transfer resulting in the formation of a double bond between C4 and C5 of the +1 sugar ring and simultaneous protonation of O4 by a general acid resulting in the cleavage of the C4-O-C1 glycosidic bond [27]. Biochemical and structural characterization of Alg17c identified a tyrosine residue (Tyr^258^) that is predicted to act as the general acid. This assertion is supported by the fact that the residue is in the correct orientation with respect to the glycosdic bond to donate a proton to O4 during the catalytic process and mutation of this tyrosine to phenylalanine abolished enzymatic activity [13]. The corresponding tyrosine in Smlt2602 (Tyr^264^) is highlighted in blue in Fig. 2 and was utilized as an anchor point to approximate the location of the tunnel-like active site in Smlt2602. Park et al. proposed that Asn^201^ and His^202^ act as the neutralizing groups, whereas Tyr^450^ acts as the general base. All three of these residues were conserved in Smlt2602 (Asn^207^, His^208^, and Tyr^455^) and are highlighted in orange in Fig. 2.

Previous studies of PLs have revealed that polar and charged residues located in the active site cleft form hydrogen bonds and salt bridges with the hydroxyl and carboxyl groups of the substrate [8]. In addition, aromatic side chains undergo C-H/π interactions with the sugar rings of the glycan substrate [28]. The summation of these interactions results in the precise positioning of the polysaccharide substrate with respect to the catalytically active residues for optimal enzymatic cleavage [29, 30]. Our own study of Smlt1473 confirmed that residues such as tyrosine, histidine, and arginine located in the active site cleft significantly influence substrate specificity and overall activity [12]. To this end, we identified eight putative substrate-binding residues (Arg^143^, Gln^153^, Leu^155^, Asn^201^, His^206^, Tyr^258^, Tyr^263^, and His^418^) highlighted in orange in Fig. 2, whose side chains are within 10 Å of the putative catalytic Tyr^264^ in the Smlt2602 homology model. Thus, a total of 12 residues were selected for analysis via site-directed mutagenesis in an effort to elucidate their role in Smlt2602, four of which are predicted to participate directly in the β-elimination mechanism (Asn^207^, His^208^, Tyr^264^, and Tyr^455^) and eight of which are predicted to bind and properly position the substrate in the active site (Arg^143^, Gln^153^, Leu^155^, Asn^201^, His^206^, Tyr^258^, Tyr^263^, and His^418^) (Fig. 2).

### Site-directed mutagenesis of putative catalytic residues indicates a tyrosine and histidine act as acid and base

**Fig. 3:**
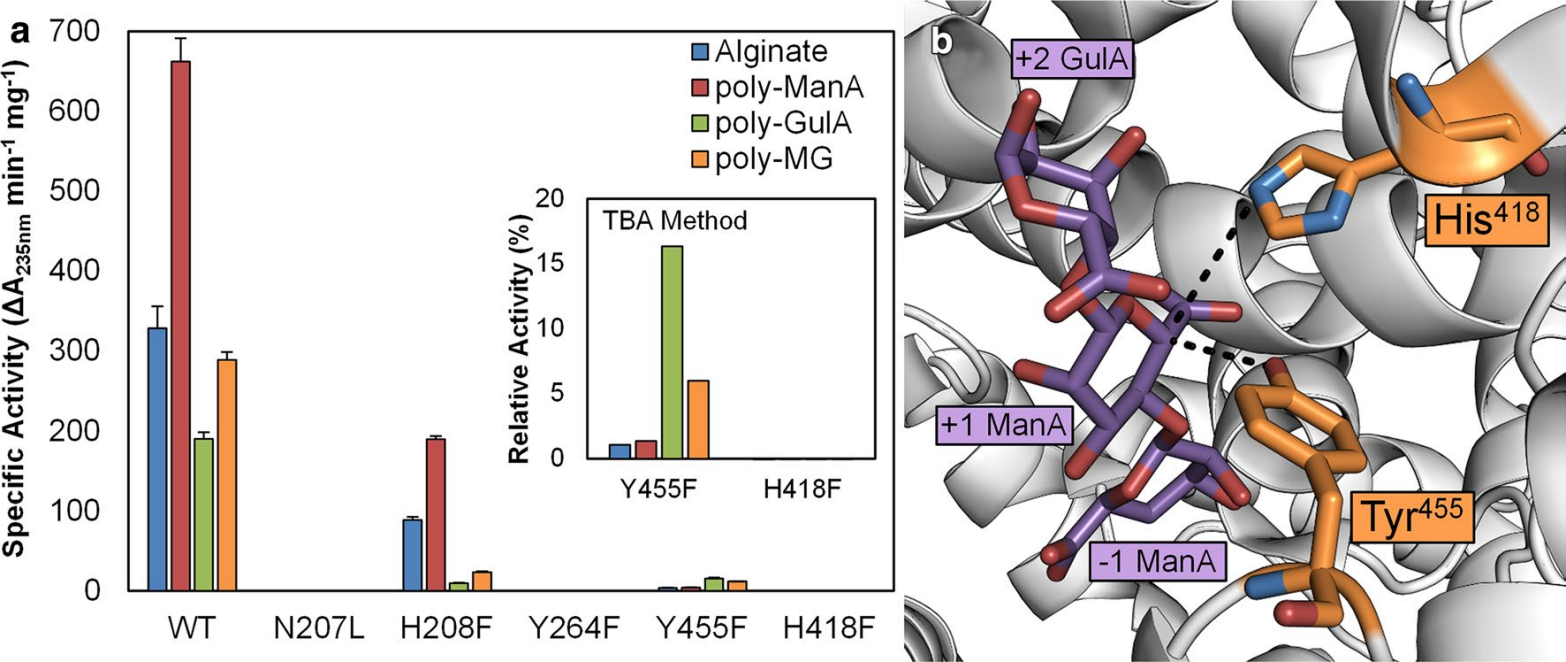
Mutagenesis of putative catalytic residues. **a** Specific activity of wild-type Smlt2602 and N207L, H208F, Y264F, H418F, and Y455F mutants against 1 mg/mL alginate-based substrates in 20 mM sodium phosphate buffer, pH 8.5. Enzyme activity was monitored by absorbance at 235 nm and confirmed via the TBA method (*inset*). For the TBA method, activity of WT against each substrate was taken to be 100 %. **b** Location of His^418^ and Tyr^455^ with respect to polysaccharide substrate, indicating each residue lies axial to the presumed location of the C5 proton (*dashed black lines*). Image generated from crystal structure of Alg17c from *S. degradans* 2–40 (Protein Data Bank 4OJZ) in complex with alginate trisaccharide (shown in *purple*). Residues in *orange* are His^418^ and Tyr^450^ of Alg17c which correspond to His^418^ and Tyr^455^ of Smlt2602 [13]

As stated previously, the β-elimination mechanism of PLs involves three steps: neutralization of the negatively charged substrate, proton abstraction by a general base, and proton donation by a general acid [27]. Despite the significant structural diversity found among PLs, the residues responsible for each of the aforementioned steps appear to be highly conserved and fall into two general categories: (i) lyases that require a divalent cation (usually Ca^2+^) to act as the neutralizing group and (ii) lyases that do not require a divalent cation and instead one or more polar or charged residues (usually arginine, asparagine, or glutamine) in close proximity to the C5 carboxylate group act as the neutralizing group. In the former, a lysine or arginine acts as the base and water as the acid. In the latter, a tyrosine or histidine acts as the base and tyrosine as the acid [8]. For example, the crystal structure of *Streptococcus pneumoniae* hyaluronate lyase in complex with substrate revealed that the amide group of Asn^239^ fulfills the role of neutralization by interacting with the glycan carboxylate group in a bidentate fashion, and Tyr^408^ and His^399^ act as the acid and base, respectively [31]. Similarly, the crystal structure of *Sphingomonas* sp. A1-III alginate lyase in complex with its substrate revealed that in addition to Asn^191^, the guanidinium group of Arg^239^ acts as the neutralizing group, with a single tyrosine residue (Tyr^246^) acting as both acid and base [32]. As discussed above, Park et al. proposed a third mechanism for Alg17c in which Asn^201^ and His^202^ act as neutralizing groups, with Tyr^258^ acting as the acid and Tyr^450^ as the base. Given that Smlt2602 and Alg17c both belong to PL-17, we hypothesize that Smlt2602 has a catalytic mechanism most similar to Alg17c, in which two tyrosine residues, Tyr^264^ and Tyr^455^ in Smlt2602, play the role of general acid and base, respectively, and Asn^207^ and His^208^ neutralize the substrate (Fig. 2). To test this hypothesis, we prepared four inactivating mutants (N207L, H208F, Y264F, and Y455F) and measured their enzymatic activity against alginate and each of its block structures (Fig. 3a). In order to confirm that any observed variations in activity are not due to an overall distortion of the active site architecture, the CD spectrum of each mutant lyase (N207L, H208F, Y264F, H419F, and Y455F) was collected and compared to WT (see Additional file 1: Figure S2 for CD spectra comparing wild-type and mutant Smlt2602). There was a less than 6 % variation in the CD signal for each lyase, indicating that the overall secondary structure of Smlt2602 was not significantly perturbed by the point mutations.

The N207L and Y264F mutants are found to be completely inactive against alginate and all three block types (Fig. 3a), whereas the H208F mutant exhibits reduced activity toward each substrate tested, with approximately 30 % WT activity against alginate and poly-ManA, and 5 and 8 % WT activity against poly-GulA and poly-MG, respectively (Fig. 3a; Table 1). Our data are in close agreement with the kinetic parameters determined for mutants of the corresponding residues in Alg17c [13] and support the hypothesis that Asn^207^ and His^208^ act as neutralizing groups and Tyr^264^ as the proton donor during the catalytic process. Given that N207L is a null mutant while H208F retains significant enzymatic activity, the Asn^207^ residue likely plays a more crucial role in the neutralization of the glycan carboxylate. Moreover, mutation of His^208^ to Phe appears to have a significantly greater detrimental effect on poly-GulA and poly-MG activity compared to alginate and poly-ManA (Fig. 3a; Table 1), implying this residue is more critical for the neutralization of the +1 sugar carboxylate group when that sugar is GulA.

The Y455F mutant exhibits significantly diminished yet measurable activity toward alginate and each of its block types (Fig. 3a). More specificity, the mutant displays less than 1 % alginate and poly-ManA activity compared to WT, yet approximately 8 and 4 % WT activity toward poly-GulA and poly-MG, with specific activities of 15.3 ± 1.4 and 11.5 ± 0.1 units/mg, respectively (Fig. 3a; Table 1). Y445F activity was confirmed via the TBA method (Fig. 3a, inset). Although the measured enzymatic rates are minimal, the fact that the Y455F mutant demonstrates any residual activity suggests that Tyr^455^ may not act as the proton acceptor in the β-elimination mechanism of Smlt2602. To that end, we consulted the Smlt2602 homology model (Fig. 2) and the Alg17c crystal structure [13] in search of an additional residue that could possibly act as the base. We located a nearby histidine in Smlt2602 (His^418^) whose corresponding histidine in Alg17c (His^415^) lies on the same side of the substrate and is within 4.3 Å of the C5 position of the +1 sugar ring, implying it could abstract the C5 proton during catalysis (Fig. 3b, dashed line). Therefore, we prepared a H418F mutant and tested its ability to cleave alginate-based substrates. We detect no increase in absorbance at 235 nm nor product formation via the TBA method, supporting the hypothesis that His^418^ is essential for catalysis (Fig. 3a).

Given the above results, we propose that Tyr^264^ acts as the proton donor in Smlt2602 and one of two scenarios is taking place with regards to the general base: (1) His^418^ acts as the proton acceptor, resulting in a catalytic mechanism similar to the hyaluronate lyase of *Streptococcus pneumoniae* [31]. In this regime, Tyr^455^ may stabilize an intermediate product of catalysis, explaining the diminished yet measurable activity of the Y455F mutant; (2) Tyr^455^ acts as the proton donor, resulting in a catalytic mechanism similar to Alg17c [13]. In this regime, the His^418^ residue may act as a surrogate, yet less efficient, proton acceptor in the absence of Tyr^264^, also explaining the diminished yet measureable activity of the Y445F mutant. In an effort to elucidate which mechanism is more likely, we considered the fact that the ionizable residue acting as the general base must be deprotonated at the pH at which the enzymatic reaction occurs. Therefore, we estimated the p*K*_*a*_ of the phenolic side of Tyr^455^ and the imidazole ring of His^418^ using PROPKA [33–36], which predicts shifts in p*K*_*a*_ resulting from interactions with the microenvironment surrounding the residue of interest [37]. Based on the Smlt2602 homology model (Fig. 2), the p*K*_*a*_ of Tyr^455^ was predicted to be shifted to 17.6, whereas the p*K*_*a*_ of His^418^ was 5.1. Similarly, the model based on the Alg17c crystal structure in complex with the alginate trisaccharide (Protein Data Bank 4OJZ) predicted the p*K*_*a*_ values of the corresponding tyrosine and histidine to be 15.4 and 3.84, respectively. Based on these estimations and given that Smlt2602 exhibited optimal activity at pH 8.5 (see Additional file 1: Figure S1 for relative activity against poly-ManA as a function of pH), it is unlikely that Tyr^455^ would be deprotonated and therefore cannot act as the general base during catalysis. Conversely, His418 would be deprotonated and able to fulfill the role of the catalytic base. Thus, we conclude that the most likely catalytic mechanism for Smlt2602 is as follows: Asn^207^ and His^208^ act as neutralizing groups, His^418^ acts as the general base, and Tyr^264^ acts as the general acid.

### Isolation of an H208F mutant that exhibits significant poly-β-d-glucuronic acid activity

Mutation of His^208^ to Phe resulted in an overall reduction in enzymatic activity toward alginate and each of its block structures (Fig. 3a; Table 1). However, the ratio of poly-ManA to poly-GulA activity shifted from 3.5 for WT to 20 for H208F, indicating that mutation of His^208^ significantly influenced the substrate specificity of Smlt2602 at the expense of overall enzymatic activity (Fig. 3a; Table 1). The *N*ϵ2 of the corresponding histidine in Alg17c (His^202^) lies 2.7 Å from the carboxylic O6A, 3.2 Å from O5, 3.9 Å from the hydroxyl O2, and 4.4 Å from the glycosidic O4 of the +1 ManA sugar. Furthermore the imidazole ring lies parallel to the +2 GulA ring at a distance of 3.8 Å [13]. Given these multiple points of contact between the substrate and the side chain of His^208^, as well as the shift in substrate specificity with respect to poly-ManA, we postulate that His^208^ of Smlt2602 plays an important role in regulating substrate specificity and merits further evaluation.

**Fig. 4:**
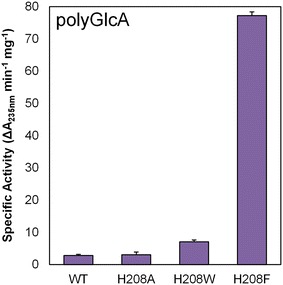
Enhanced poly-GlcA activity via mutagenesis of His^208^ residue. Specific activity of WT and H208A, H208W, H208F mutants against 1 mg/mL poly-GlcA in 20 mM sodium phosphate buffer, pH 8. Enzymatic activity was monitored by change in absorbance at 235 nm. All reactions were performed in triplicate and error is reported as standard deviation

Remarkably, substrate specificity analysis of H208F revealed that the mutant lyase exhibited significant enzymatic activity toward poly-GlcA, a non-alginate-based substrate (Fig. 4; Table 1). To our knowledge, no other characterized PL-17 lyase has been shown to cleave poly-GlcA [25]. The specific activity of H208F against poly-GlcA (77.2 ± 1.2 units/mg) is a 28-fold increase over WT poly-GlcA activity (2.8 ± 0.4 units/mg) and is within an order of magnitude of WT alginate activity (328.3 ± 27.0) (Fig. 4b; Table 1). Moreover, the H208F poly-GlcA activity is on the same order of magnitude as other poly-GlcA specific lyases [5, 38, 39]. Note that the optimal pH of activity was slightly shifted from pH 8.5 for alginate-based substrates to pH 8 for poly-GlcA (see Additional file 1: Figure S3 for relative activity of Smlt2602 H208F mutant versus pH against poly-GlcA). Substrate-dependent variations in optimal pH have been demonstrated before in PLs and may be indicative of slightly different binding of each substrate [5].

In the interest of determining, if the novel poly-GlcA activity was specific for the phenylalanine mutation or if any mutation of His^208^ would recapitulate the H208F result, we generated two other mutant lyases (H208A and H208W) and measured their ability to digest poly-GlcA. H208A was indistinguishable from WT, whereas H208 W demonstrated a minute twofold increase in activity (7.1 ± 0.6 units/mg) (Fig. 4). Thus our results indicate that the novel poly-GlcA activity displayed by mutation of His^208^ is at least partially specific to the phenylalanine substitution (Fig. 4; Table 1).

### Size-exclusion chromatography analysis of carbohydrate products formed by Smlt2602 and H208F revealed an exolytic mode of action

**Fig. 5:**
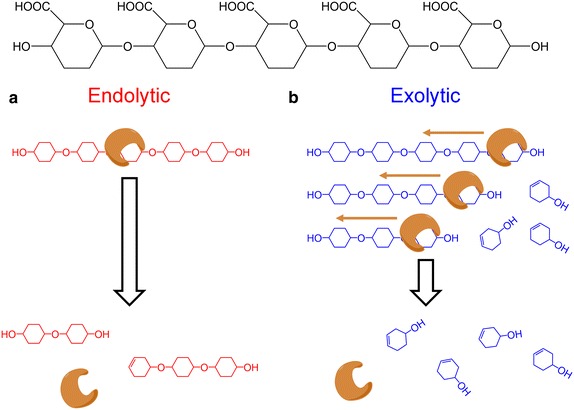
Endolytic and exolytic modes of polysaccharide cleavage. **a** Endolytic enzyme binds to an internal site on the polysaccharide, cleaves the *O*-glycosidic bond via β-elimination, and detaches, resulting in products of various sizes. **b** Exolytic enzyme binds to one end of the polysaccharide, undergoes β-elimination, clipping off an unsaturated monomer while simultaneous sliding down the polysaccharide to the next cleavable bond. The process repeats until the entire polysaccharide has been processed into unsaturated monomers

PLs are known to act on polyuronides endolytically, exolytically, or a combination of both. Endolytic lyases bind to the polysaccharide substrate internally, cleave one glycosidic bond, and detach, resulting in the simultaneous accumulation of multiple unsaturated products of various sizes [38, 40–42] (Fig. 5a). Exolytic enzymes bind to the nonreducing end of the polysaccharide substrate and cleave off mono-, di-, or trisaccharides until the entire macromolecule has been digested, resulting in the accumulation of predominantly one unsaturated product, with little to no accumulation of differently sized oligosaccharides [11, 42–44] (Fig. 5b). The hyaluronate lyases of *Streptococcus* sp. exhibit a hybrid mechanism, in which hyaluronan is digested by an initial random endolytic cleavage and subsequent exolytic processing into disaccharides [31]. Multiple PL-17 lyases, including Alg17c, have been shown to act exolytically on their alginate substrates, reducing the polysaccharide to unsaturated monomers [45–47]. Due to Smlt2602 belonging to the same family and sharing 50 % sequence identity with Alg17c, we predicted that Smlt2602 is an exolytic lyase.

**Fig. 6:**
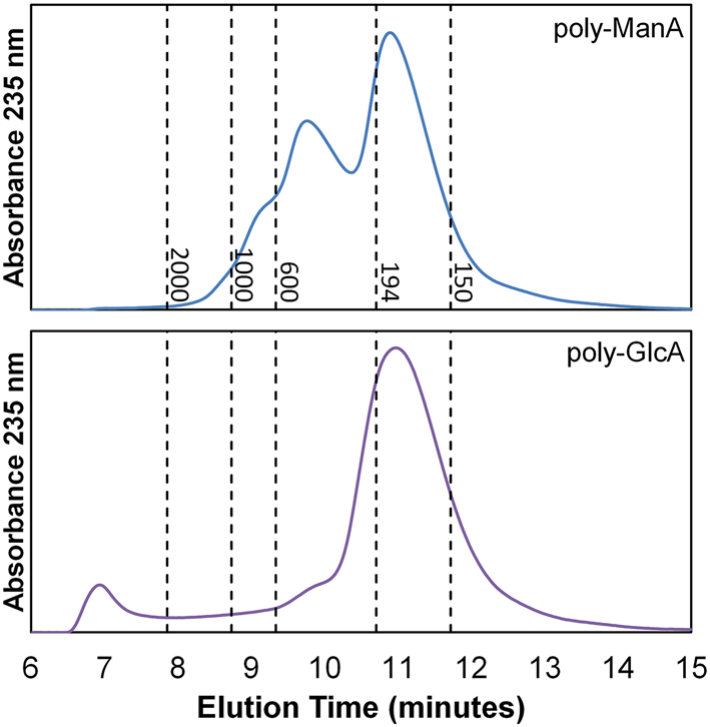
Analysis of unsaturated sugar products formed by WT against poly-ManA (*top*) and H208F mutant against poly-GlcA (*bottom*). HPLC chromatograms of products formed 6 h after addition of enzyme. Dashed vertical lines indicate the elution time of molecular weight standards (PEG2000, PEG1000, PEG600, d-glucuronic acid, and xylose from left to right)

To test this hypothesis, the carbohydrate products formed during Smlt2602 cleavage of both poly-ManA and poly-GlcA were analyzed by size-exclusion chromatography (Fig. 6). Briefly, poly-ManA and poly-GlcA was digested with WT and H208F, respectively. After 6 h, the reaction mixture was separated on a TSKgel SuperOligoPW column and unsaturated products were detected by monitoring absorbance at 235 nm. A series of molecular weight standards were also run to assist in determining the molecular weight of the sugar products (Fig. 6, dashed vertical lines). The most prominent peak observed for poly-ManA and poly-GlcA eluted between the peaks of d-glucuronic acid and xylose, which have molecular weights of 194 and 150 Da, respectively. A calibration curve was generated from the molecular weight standards, and the molecular weight of this most prominent species was estimated to be 166 Da, in close agreement with the molecular weight of ManA and GlcA unsaturated monomers (176 Da). This is consistent with the exolytic mode of action observed in other PL-17 lyases.

As stated previously, exolytic cleavage usually results in the accumulation of a single sugar species. A single species was detected for the poly-GlcA run (Fig. 6 bottom), whereas an additional peak of lower abundance was detected for the poly-ManA run. This second species was estimated to have molecular weight of 378 Da, in close agreement with the molecular weight of a disaccharide comprised a saturated and unsaturated uronic acid (368 Da) (Fig. 6 top). Given that WT demonstrates the lowest activity toward poly-G (Fig. 1b; Table 1), it is possible this secondary product is a Δ-GulA dimer. Regardless, the accumulation of unsaturated monomers is indicative of an exolytic mode of action for Smlt2602 and H208F against poly-ManA and poly-GlcA, respectively. To our knowledge, this is the first characterized bacterial PL with exolytic alginate and poly-GlcA activity [25].

### Kinetic parameters of Smlt2602 and H208F

In order to evaluate the effect, the His^208^ to Phe mutation had on substrate binding and product conversion, the Michaelis constant (*K*_*M*_) and the turnover number (*k*_CAT_) of WT and H208F were determined for alginate, poly-ManA, poly-GulA, poly-MG, and poly-GlcA (Table 2). The two parameters are dependent on one another and their ratio (*k*_CAT_/*K*_*M*_) is considered a measure of enzyme efficiency [30, 32]. Note that although *K*_*M*_ may approximate substrate affinity, it is also affected by other rate and equilibrium constants, and therefore *k*_CAT_/*K*_*M*_ is used as a descriptive parameter for substrate binding [48]. As can be seen is Table 2, the *k*_CAT_ values for WT trend with the specific activity measured for each substrate (Fig. 1b), with poly-ManA having the highest turnover rate (62.2 s^−1^) and alginate, poly-GulA, and poly-MG all having approximately half that rate (34.8, 34.6, and 22.2 s^−1^, respectively). WT also exhibited residual turnover of poly-GlcA (0.4 s^−1^). The *K*_*M*_ values of WT for the alginate-based substrates also followed a similar trend, with poly-ManA having the lowest *K*_*M*_ value (0.41 mM), reflective of the highest substrate affinity. Interestingly, the *K*_*M*_ value of poly-GulA (5.99 mM) is at least fourfold higher than any other substrate, including poly-GlcA. Thus, the enzyme efficiency of WT toward poly-GulA (5.8 mM/s) was the lowest for all the alginate-based substrates and is indicative of poor poly-GulA binding. Overall, our results demonstrate that WT is an alginate-specific lyase, with highest substrate affinity and product turnover for poly-ManA (Table 2).

**Table 2:** Substrate specificity ratios of mutant lyase

	ManA/GulA	ManA/MG
WT	3.5 ± 0.1	2.3 ± 0.1
R143L	2.6 ± 0.1	1.5 ± 0.1
Q153A	15.1 ± 1.5	6.2 ± 0.3
Q153N	3.9 ± 0.1	3.0 ± 0.3
L155A	21.1 ± 0.6	6.7 ± 0.1
L155N	12.1 ± 0.8	3.9 ± 0.1
N201L	6.6 ± 0.6	3.6 ± 0.1
H206F	2.6 ± 0.1	2.0 ± 0.1
Y258F	3.9 ± 0.2	2.7 ± 0.2
Y263F	15.7 ± 1.7	6.2 ± 0.6

Ratios were calculated by dividing the poly-ManA activity of each mutant over the poly-GulA or poly-MG activity

The H208F mutant demonstrated decreases in *k*_CAT_ for all alginate-based substrates relative to WT (Table 2), in agreement with the measured specific activities for each substrate (Fig. 3a). Furthermore, H208F displayed a 15-fold increase in turnover rate for poly-GlcA (Table 2), reflective of the 28-fold increase in specific activity relative to WT (Fig. 4). This increase in *k*_CAT_ was coupled with a decrease in *K*_*M*_ from 1.4 mM for WT to 0.57 for H208F, on pair with the binding of poly-ManA (0.41 mM) for each enzyme. The increase in turnover rate and decrease in Michaelis constant resulted in 35-fold increase in enzyme efficiency toward poly-GlcA, from 0.3 for WT to 10.6 for H208F. This value for enzyme efficiency is on the same order of magnitude as WT efficiency toward alginate (Table 2) and within one order of magnitude of the value for the highly active, poly-GlcA specific, and endolytic lyase Smlt1473 [5]. Overall, our results demonstrate that H208F is multifunctional PL, with significant binding and exolytic turnover of both alginate- and glucuronan-based substrates (Table 2), which, to our knowledge, is unprecedented for PLs [25].

It is interesting to consider the structural differences between the GlcA and ManA sugar residues in the context of explaining the increase in poly-GlcA activity observed for the H208F mutant. The only structural difference between the ManA and GlcA monomers is the positioning of the C2 hydroxyl group. In ManA, the hydroxyl group points up, axial to the sugar ring, whereas in GlcA, it points down—equatorial to the ring [8]. As stated previously, the *N*ϵ2 of the histidine in Alg17c corresponding to His^208^ in Smlt2602 lies 3.9 Å away from the C2 hydroxyl group of the +1 ManA sugar and the two atoms likely form a hydrogen bond. This bond would be disrupted upon mutation to phenylalanine and may account for the reduced enzymatic efficiency of the H208F mutant toward poly-ManA. Given that ManA and GlcA are C2 epimers, the *N*ϵ2 of His^208^ would be unlikely to form a hydrogen bond with the C2 hydroxyl of the +1 sugar due to the hydroxyl group pointing in the opposite direction relative to the sugar ring. Therefore, the His^208^ mutation would not affect binding of poly-GlcA at this position. This, of course, does not explain the novel poly-GlcA activity of H208F, but it is an example of how the same point mutation can have divergent effects of two chemically related substrates. A high-resolution crystal structure of Smlt2602 and H208F in complex with poly-ManA and poly-GlcA would be required to fully elucidate the mechanism behind the enhanced poly-GlcA activity of H208F.

### Substrate analysis of additional putative substrate-binding mutants

Residues located in the active site cleft but not directly involved in the β-elimination mechanism are thought to participate in substrate binding and alignment with respect to the catalytically active residues to allow for optimal cleavage of the glycosidic bond. Mutation of such residues in the endolytic lyase Smlt1473 resulted in significant changes in substrate specificity and overall enzyme activity and thus could be applied to designing highly active and specific endolyases [12]. In an effort to explore whether the same principle applies for exolytic lyases and given that the H208F mutant exhibited significant poly-GlcA activity that was absent from WT, we mutated seven additional putative substrate-binding residues (Arg^143^, Gln^153^, Leu^155^, Asn^201^, His^206^, Tyr^258^, Tyr^263^, and His^418^), identified via homology modeling (Fig. 2). A total of ten mutants were prepared (R143L, Q153A, Q153N, L155A, L155N, N201L, H206F, Y258F, Y263F, and H418F) with some residues being mutated to two different amino acids. Although all of the additional mutants, with the exception of N201L against poly-ManA, displayed an overall decrease in activity that was independent of substrate, a number of them did exhibit significant changes in substrate specificity toward alginate and its three block types (Table 1).

**Fig. 7:**
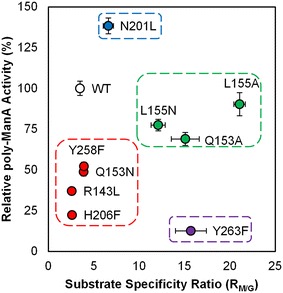
Substrate specificity and overall activity of mutant lyases. The substrate specificity ratio (R_M/G_), defined as the ratio of poly-ManA activity over poly-GulA activity, with respect to overall poly-ManA activity for each mutant lyase. Mutants were found to group in four general categories as discussed in the text. Note that 100 % activity was taken as the activity of WT against poly-ManA

Given that WT is most active against poly-ManA, we analyzed the mutants by calculating the ratio of poly-ManA activity to poly-GulA activity or poly-MG, designated “R_M/G_” and “R_M/MG_,” respectively (Table 3). All R_M/G_ and R_M/MG_ values were greater than unity, and therefore, all mutant lyases remained most active toward poly-ManA compared to poly-GulA and poly-MG. The R_M/G_ and R_M/MG_ values for WT are 3.5 and 2.3, respectively. An increase in either value over WT represented a shift in substrate specificity toward poly-ManA over the other substrate. Four of the mutants (R143L, Q153N, H206F, and Y258F) displayed minimal changes in R_M/G_ and R_M/MG_ relative to WT. The rest exhibited significant increases in both R_M/G_ and R_M/MG_, indicating that the point mutations were causing the mutant lyases to favor ManA–ManA linkages, which are the predominant bond found in poly-ManA, over GulA–GulA, ManA-GulA, and GulA-ManA linkages found in the other two substrates (Table 3). Given that R_M/G_ and R_M/MG_ trended together, we focused our analysis on comparing the R_M/G_ ratio to the overall poly-ManA activity (Fig. 7).

**Table 3:** Substrate specificity of Smlt2602 mutants

	Specific activity (ΔA_235nm_ min^−1^ mg^−1^)				
	Alginate	Poly-ManA	Poly-GulA	Poly-MG	Poly-GlcA
WT	328.3 ± 27.0	661.9 ± 29.1	190.3 ± 8.3	289.3 ± 9.8	2.8 ± 0.4
Possible involvement in β-elimination mechanism					
Y455F	3.6 ± 0.3	4.0 ± 0.4	15.3 ± 1.4	11.5 ± 0.1	0.2 ± 0.1
Enhanced poly-GlcA activity					
H208A	48.2 ± 3.5	83.2 ± 4.2	6.3 ± 0.8	3.6 ± 0.4	3.1 ± 0.8
H208W	185.3 ± 11.8	490.8 ± 4.1	48.5 ± 1.0	117.1 ± 7.8	7.1 ± 0.6
H208F	88.4 ± 4.0	189.7 ± 4.1	9.5 ± 0.8	23.3 ± 1.0	77.2 ± 1.2
Additional putative substrate-binding residues					
R143L	224.1 ± 13.9	245.4 ± 15.3	95.5 ± 2.5	159.0 ± 9.2	1.1 ± 0.3
Q153A	129.0 ± 3.5	456.4 ± 26.4	30.3 ± 3.5	73.6 ± 4.4	0.2 ± 0.1
Q153 N	113.8 ± 9.9	323.7 ± 17.1	83.4 ± 2.6	108.7 ± 10.7	0.9 ± 0.2
L155A	158.5 ± 9.5	597.6 ± 46.9	28.4 ± 0.9	89.6 ± 1.4	0.3 ± 0.1
L155 N	125.6 ± 12.6	512.7 ± 23.2	42.4 ± 2.9	131.9 ± 4.8	0.3 ± 0.2
N201L	380.1 ± 9.2	915.8 ± 31.9	138.6 ± 12.9	255.3 ± 9.9	3.1 ± 0.7
H206F	73.9 ± 2.3	147.8 ± 3.9	57.0 ± 2.9	73.2 ± 0.2	0.4 ± 0.1
Y258F	173.4 ± 9.1	346.2 ± 16.1	88.7 ± 6.0	127.7 ± 9.5	0.5 ± 0.1
Y263F	36.1 ± 0.4	83.5 ± 1.7	5.3 ± 0.6	13.5 ± 1.4	0.2 ± 0.1

Purified wild-type and mutant Smlt2602 was added to 1 mg/mL of each substrate in 20 mM sodium phosphate buffer at pH 8.5 for alginate-based polysaccharides and pH 8 for poly-GlcA. Enzymatic activity was monitored by absorbance at 235 nm. In addition to the substrates listed below, each enzyme was tested against poly-GalA, heparin, heparan sulfate, and hyaluronic acid, with no detectable activity. All reactions were performed in triplicate, and error is reported as standard deviation

The mutant lyases were found to group into four general categories, colored coded in Fig. 7. The first category comprised R143L, Q153N, H206F, and Y258F (Fig. 7, red). These mutants displayed minute changes in substrate specificity compared to WT and exhibited ≤52 % overall poly-ManA activity. Thus, mutation of these residues impedes either binding or catalysis, independent of substrate. The second category comprised Q153A, L155A, and L155 N (Fig. 7, green). The overall poly-ManA of each of these mutants was ≥68 %, and the R_M/G_ values were ≥12, indicating these lyases were highly selective for poly-ManA with overall activity on pair with WT. The third category comprised Y263F (Fig. 7, purple) which displayed the lowest overall poly-ManA activity (12.6 %) and R_M/G_ value of 15.7, indicating the mutant is highly selective for poly-ManA, but catalysis is severely impeded. Given its close proximity to the catalytic acid (Tyr^264^), it is likely that Tyr^263^ plays an indirect role in catalysis (Fig. 2). The fourth category comprised N201L (Fig. 7, blue), the only mutant to exhibit an increase in overall poly-ManA activity (138 %). Furthermore, the R_M/G_ value was 6.6, almost twice that of WT. Therefore, this mutant is both highly active and specific for poly-ManA. Overall our results show that it is possible to modulate the substrate specificity of Smlt2602 through mutation of residues located near the catalytic core.

## Conclusions

In summary, we describe the characterization of a PL-17 polysaccharide lyase from *Stenotrophomonas maltophilia* K279a which exolytically degrades alginate. Through homology modeling we identified a key residue (His^208^) thought to be involved in substrate binding and upon mutation, we were able to introduce novel exolytic poly-GlcA activity into the lyase. Numerous exolytic polysaccharide lyases have been described which degrade alginate [23], poly-GalA [49], hyaluronan [31], heparan sulfate [50], and chondroitin sulfate [51]. To our knowledge, no lyase has even been described that exolytically digests both alginate and poly-GlcA, and as such, Smlt2602 could be utilized to efficiency digest algae polysaccharides into fermentable monomers for biofuel production. Furthermore, our work demonstrates the possibility of engineering substrate specificity into lyases, which has applications in energy and chemical production, but in the design of glycan-based chemicals as well.

## Additional file

10.1186/s13068-016-0455-8This file contains additional figures (S1, S2, S3) referenced in the manuscript; **Figure S1**. Optimal pH of WT against poly-ManA. **Figure S2**. Circular dichroism spectrum of wild-type and mutant Smlt2602. **Figure S3**. Optimal pH of H208F mutant against poly-GlcA.

## Abbreviations

ManA: d-mannuronic acid
GulA: l-guluronic acid
GlcA: d-glucuronic acid
PL: polysaccharide lyase
WT: wild-type
poly-GalA: poly-α-d-galacturonic acid
