# Supplementary material for: Engineering broad-spectrum digestion of polyuronides from an exolytic polysaccharide lyase
Source: Biotechnol Biofuels. 2016 Feb 24;9:43. doi: 10.1186/s13068-016-0455-8 (PMC4765187; doi:10.1186/s13068-016-0455-8)
Supplement: Supplementary file 1 — 10.1186/s13068-016-0455-8This file contains additional figures (S1, S2, S3) referenced in the manuscript; Figure S1. Optimal pH of WT against poly-ManA. Figure S2. Circular dichroism spectrum of wild-type and mutant Smlt2602. Figure S3. Optimal pH of H208F mutant against poly-GlcA. [file 13068_2016_455_MOESM1_ESM.docx]

**Supplemental Information**

**Engineering broad-spectrum digestion of polyuronides from an exolytic polysaccharide lyase**

Logan C. MacDonald^†^, Elizabeth B. Weiler^†^, and Bryan W. Berger^†§*^

Additional Figure S1


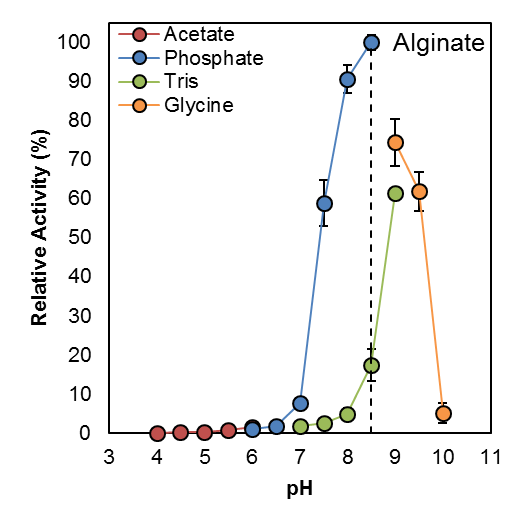


**Additional Figure S1: Optimal pH of WT against poly-ManA.** The pH of each reaction was maintained by the indicated buffer at 20 mM total ionic strength. Enzyme activity was monitored by TBA method. All reactions were performed in triplicate and error is reported as standard deviation.

Additional Figure S2


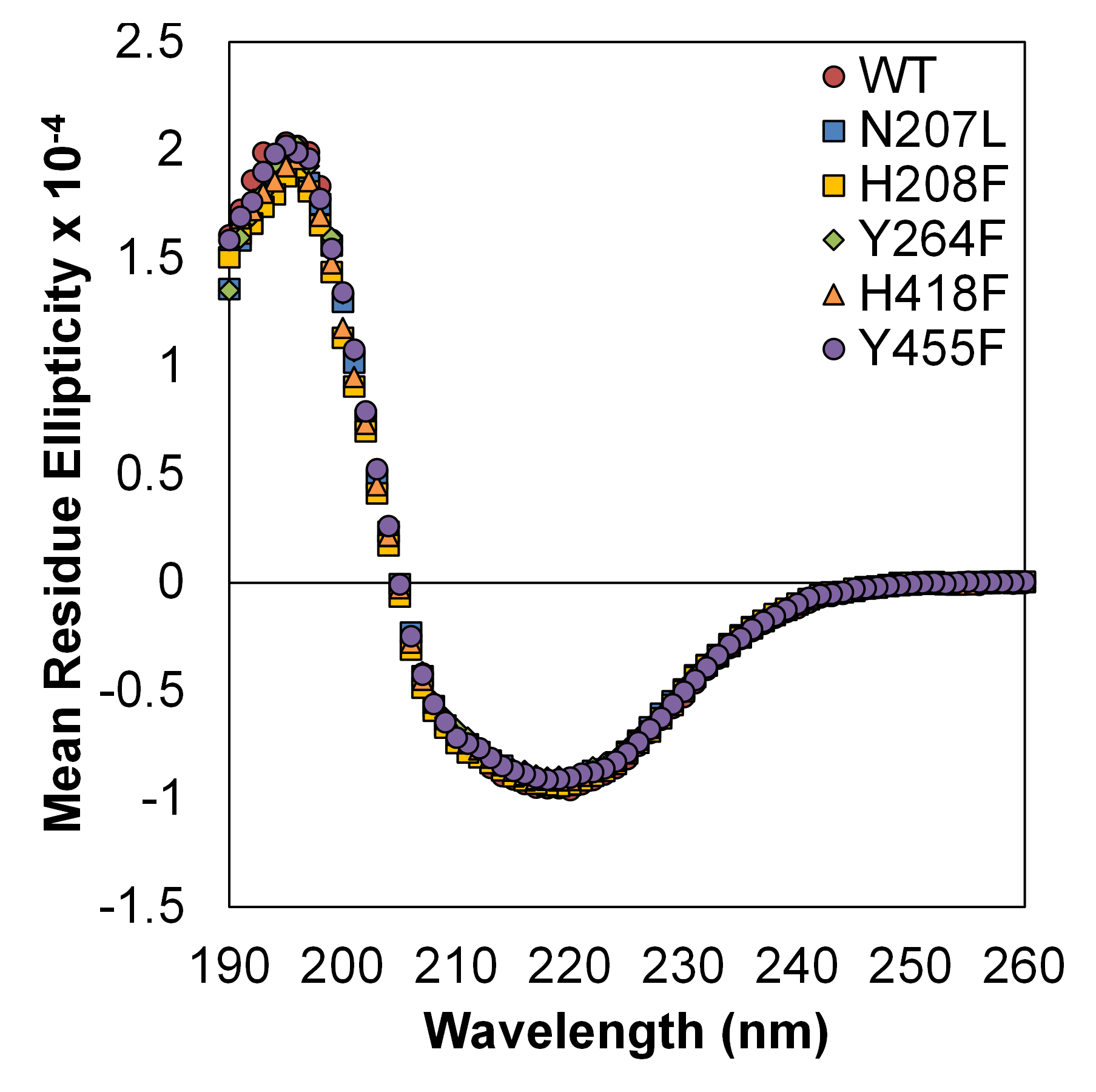


**Additional Figure S2: Circular dichroism spectrum of wild-type and mutant Smlt2602.** All protein samples were measured at 250 µg/mL in 20 mM sodium phosphate buffer, pH 8.5.

Additional Figure S3


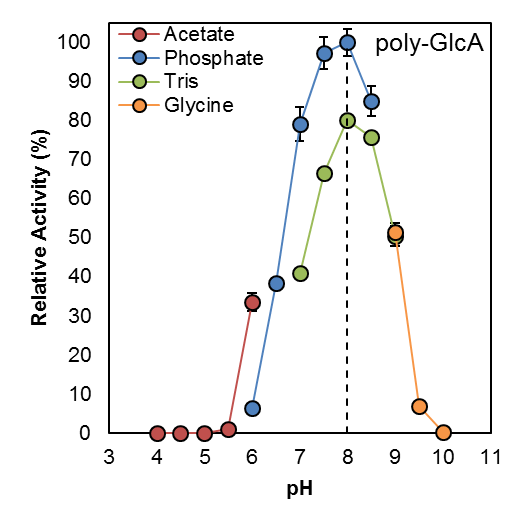


**Additional Figure S3: Optimal pH of H208F mutant against poly-GlcA.** The pH of each reaction was maintained by the indicated buffer at 20 mM total ionic strength. Enzyme activity was monitored by TBA method. All reactions were performed in triplicate and error is reported as standard deviation.
